# Hourly potential evapotranspiration at 0.1° resolution for the global land surface from 1981-present

**DOI:** 10.1038/s41597-021-01003-9

**Published:** 2021-08-24

**Authors:** Michael Bliss Singer, Dagmawi Teklu Asfaw, Rafael Rosolem, Mark O. Cuthbert, Diego G. Miralles, David MacLeod, Edisson Andres Quichimbo, Katerina Michaelides

**Affiliations:** 1 School of Earth and Environmental Sciences, Cardiff University, Cardiff, CF10 3AT United Kingdom; 2 Water Research Institute, Cardiff University, Cardiff, CF10 3AX United Kingdom; 3 Earth Research Institute, University of California Santa Barbara, Santa Barbara, CA 93106 USA; 4 School of Geographical Sciences, University of Bristol, Bristol, BS8 1SS United Kingdom; 5 Department of Civil Engineering, University of Bristol, BS8 1TR Bristol, United Kingdom; 6 Cabot Institute for the Environment, University of Bristol, Bristol, BS8 1QU United Kingdom; 7 School of Civil and Environmental Engineering, The University of New South Wales (UNSW), Sydney, Australia; 8 Hydro-Climate Extremes Lab (H-CEL), Ghent University, Ghent, 9000 Belgium

**Keywords:** Hydrology, Hydrology

## Abstract

Challenges exist for assessing the impacts of climate and climate change on the hydrological cycle on local and regional scales, and in turn on water resources, food, energy, and natural hazards. Potential evapotranspiration (PET) represents atmospheric demand for water, which is required at high spatial and temporal resolutions to compute actual evapotranspiration and thus close the water balance near the land surface for many such applications, but there are currently no available high-resolution datasets of PET. Here we develop an hourly PET dataset (hPET) for the global land surface at 0.1° spatial resolution, based on output from the recently developed ERA5-Land reanalysis dataset, over the period 1981 to present. We show how hPET compares to other available global PET datasets, over common spatiotemporal resolutions and time frames, with respect to spatial patterns of climatology and seasonal variations for selected humid and arid locations across the globe. We provide the data for users to employ for multiple applications to explore diurnal and seasonal variations in evaporative demand for water.

## Abstract (summary)

**Table:** 

Measurement(s)	evapotranspiration
Technology Type(s)	computational modeling technique
Factor Type(s)	temporal interval
Sample Characteristic - Environment	climate system • hydrological process
Sample Characteristic - Location	global

Machine-accessible metadata file describing the reported data: 10.6084/m9.figshare.15060387

## Background & Summary

Terrestrial evaporative demand is a key climatic quantity that controls the removal of water from land back to the atmosphere and is of paramount importance to the global water cycle^1–4^. Theoretical evaporative demand is often formally quantified as potential evapotranspiration (PET), which is required to calculate actual evapotranspiration and close the water balance for numerous applications ranging from water resources to agriculture to natural hazards to climate change impact analysis. PET is computed based on the energy, aerodynamics, and saturation demand in the atmosphere available for evaporation near the Earth’s surface, whether it be from open water bodies, soils, or the surface of vegetation (either as a result of interception or transpiration), unconstrained by water availability. While multiple definitions of PET exist in literature (see e.g.^5^), there exist widely accepted conventions for calculation of PET from commonly available climate variables including solar radiation, relative humidity, air temperature, and wind^6–11^. However, there is currently no available global PET dataset developed by conventional methods (e.g., Penman-Monteith, Priestley-Taylor) over recent decades with the combined high spatial and temporal resolutions required as input to many environmental models and analyses.

Spatial differences and temporal changes in PET may have profound impacts on terrestrial near-surface water fluxes and water storage (e.g., runoff, soil moisture, groundwater, evapotranspiration), which in turn have important implications for plant/crop maintenance/growth, ecology, natural hazards, and water resources for human society^1,8^. For example, in drylands, which comprise ~41% of the global land surface and a third of the world’s population^12^, the synchronicity and temporal offsets between rainfall and evaporative demand over diurnal and seasonal cycles can have major implications for the water balance and plant-available water, especially since antecedent soil moisture prior to rainstorms is typically low^11,13–17^. Correspondingly, the temporally evolving balance between precipitation, antecedent moisture, and PET affects societally relevant outcomes of the hydrologic cycle in drylands and many humid lands^18^ such as agricultural yields, the availability of fodder vegetation for livestock, the risk of flash floods and droughts, and access to groundwater^19–22^, and it could impact landscape evolution^23,24^.

A key rationale for high temporal resolution in PET is that plant water stress occurs mainly around midday and early afternoon, so intra-daily variability in PET is needed to characterize root-zone water availability, plant water uptake, and water stress^25–28^, and it can also generate feedbacks between soil moisture and rainfall^18,29,30^. Therefore, characterization of the driving atmospheric demand (PET) at high (sub-daily) resolution, which may vary dramatically across a region and between regions, is required to assess the energy balance and its controls on soil moisture, plant transpiration, land surface evaporation, and precipitation feedbacks over a day, seasonally, or even over years to decades.

High temporal resolution PET datasets do exist at specific geographical locations, but these are typically derived from *in situ* climate variables collected from limited networks (e.g., meteorological stations, eddy flux towers^5^), and are therefore neither global nor gridded. There are also existing gridded global PET datasets over land that are based on interpolation of meteorologic station data (e.g.^31^) and/or based on estimation from remote sensing data (e.g.^32^) at temporal resolutions ranging from monthly down to several days. Although each of these datasets is useful for certain purposes, there is no currently available global PET product for multiple decades in the recent historical past, based on either the Penman-Monteith or Priestly-Taylor methods, which can capture both diurnal (sub-daily) and seasonal variations in evaporative demand.

As atmospheric temperature has risen across most of the globe due to anthropogenic forcing, PET may also increase for many locations^11,33,34^, though declines in wind speed may balance out these increases^35^. However, quantification of PET changes, their timing relationships with precipitation, and their impact on the hydrological cycle is limited by a lack of high spatial and temporal resolution information across the globe. This missing dataset thwarts efforts to close the water balance in point-based calculations and to parameterize hydrological models and land surface models at catchment to regional scales, especially in areas with very high aridity. A new, high-resolution dataset on PET is essential to quantitatively evaluate a range of climate impacts^36–38^, particularly within dryland regions^18^. We are not arguing, however, that *in situ* data be replaced by gridded data. Rather, we see *in situ* data collection efforts as critical for understanding how global warming is reflected within the key parameters that comprise PET (e.g., wind speed), which will support improvements in global climate modeling and the products that emerge from it.

To fill the spatial and temporal shortcomings *in situ* data networks and existing gridded datasets, we have developed an hourly dataset of PET (hPET), which we also provide as a daily aggregation (dPET), the details of which are described below. The hPET dataset is computed using meteorological variables output from the ERA5-Land dataset recently released by the European Centre for Medium-Range Weather Forecasts (ECMWF). The publicly available ERA5-Land dataset (https://cds.climate.copernicus.eu/cdsapp#!/dataset/reanalysis-era5-land?tab=overview) is based on the latest ERA5 meteorological reanalysis as forcing data to drive the ECMWF’s land surface model Scheme for Surface Exchanges over Land incorporating land surface hydrology (H-TESSEL). ERA5-Land provides global output of meteorological data for 1981-present at hourly resolution over a spatial grid of 0.1° or 9 km at the equator.

## Methods

PET was calculated based on the standard method for computing reference evapotranspiration developed by the Food and Agriculture Organization (FAO)^6^, although we acknowledge that there are other atmosphere-only methods used to compute PET^5^. This method requires seven climate variables to compute PET for each location including zonal and meridional components of wind speed, air and dew point temperature, net solar and net thermal components of radiation, and atmospheric pressure at the Earth’s surface. We downloaded the complete global land surface dataset of ERA5-Land reanalysis meteorological variables (and their units) required to estimate PET:

1. 10 m u-component (zonal) of wind speed [m s^−1^]
2. 10 m v-component (meridional) of wind speed [m s^−1^]
3. 2 m dew point temperature [K]
4. 2 m air temperature [K]
5. surface net solar radiation [J m^−2^]
6. surface net thermal radiation [J m^−2^]
7. atmospheric surface pressure [Pa]

Hourly potential evapotranspiration (hPET) was calculated via the FAO’s Penman-Monteith equation for reference crop evapotranspiration described in (ref. ^6^). The equation is developed to compute reference evapotranspiration rate (ET_0_) based on a hypothetical reference crop with an assumed height of 0.12 m having a surface resistance of 70 s m^−1^, closely resembling the evaporation of an extension surface of green grass of uniform height, actively growing and adequately watered. Notice, however, that we use surface net solar radiation for meteorological forcing. As a result, our estimates will vary slightly from the assumed 0.23 constant albedo in reference evapotranspiration via the FAO method. This is one of the reasons we refer to our product as potential rather than reference evapotranspiration. Since PET is a notional variable, different definitions are encountered in the literature, and frequently, the concepts of ET_0_, PET, and atmospheric demand for water are used interchangeably^5,11^. In this study we use ET_0_, which can be uniformly applied across the globe to obtain a standardized and well-accepted metric of PET. PET can be computed on hourly timescales for each hour (*t*) and for each pixel location (*x*) as^6^:

1 $$hPE{T}_{x,t}=\frac{0.408\Delta ({R}_{n}-G)+\gamma \left(\frac{37}{{T}_{a}+273}\right){u}_{2}({e}_{s}-{e}_{a})}{\Delta +\gamma (1+0.34{u}_{2})}$$

where *R*_*n*_ is hourly net radiation (MJ m^−2^), *G* the soil heat flux (MJ m^−2^), *γ* is the psychrometric constant (kPa °C^−1^), ∆ is slope of saturation vapour pressure curve (kPa °C^−1^), *T*_*a*_ is hourly air temperature (°C) after converting from ERA5-Land temperature in K, *e*_*s*_ is hourly saturation vapour pressure (kPa), *e*_*a*_ is actual hourly vapour pressure (kPa), and *u*_2_ is the hourly wind speed (m s^−1^) at 2 m above the land surface. We have converted the wind speed output from ERA5-Land for the original 10 m height above the land surface to the required 2-m value based on a logarithmic velocity profile above a short grass surface^6^ as:

2 $${u}_{2}={u}_{z}\left(\frac{4.87}{ln(67.8z-5.42)}\right)$$

where *u*_*z*_ is the wind speed at *z* height above the surface computed as ${u}_{z}=\sqrt{{u}^{2}+{v}^{2}}$, for which wind speed data are available (*z* = 10 m in our case). This assumption may lead to errors in cases where the shape of velocity profile does not follow this form due to non-neutral conditions. Also, since we are making our calculation on an hourly basis, *e*_*s*_ and *e*_*a*_ are computed based on the Tetens equation^39^ as a function of hourly air (*T*_*a*_) and dew point temperature (*T*_*dew*_) in °C after converting from K, respectively, as:

3 $${e}_{s}=0.6108\exp \left(\frac{17.27\ast {T}_{a}}{{T}_{a}+237.3}\right)$$

4 $${e}_{a}=0.6108\exp \left(\frac{17.27\ast {T}_{dew}}{{T}_{dew}+237.3}\right)$$

The slope of saturation vapour pressure curve (*∆*) is calculated as:

5 $$\Delta =\frac{4098{e}_{s}}{{({T}_{a}+237.3)}^{2}}$$

And the psychrometric constant (*γ*) is calculated as:

6 $$\gamma =\frac{{C}_{p}\ast P}{\varepsilon \ast \lambda }$$

where *P* is atmospheric pressure, *C*_*p*_ is the air’s specific heat at constant pressure based on the ideal gas law with a value of 1.013 × 10^−3^ MJ kg^−1^ per °C, ε is the ratio of the molecular weight of water vapor to that of dry air, or 0.622, and *λ* is the latent heat of vaporization, a constant value of 2.45 MJ kg^−1^.

Net radiation in MJ m^−2^, *R*_*n*_, is estimated from net solar radiation (*R*_*s*_) and net thermal radiation (*R*_*t*_) as:

7 $${R}_{n}={R}_{s}-{R}_{t}$$

Note that since *R*_*t*_ is provided from ERA5-Land as a negative value, we compute this equation as: *R*_*n*_ = *R*_*s*_ + *R*_*t*_. Soil heat flux, *G* is estimated separately for daytime and nighttime as:

8 $$G=\left\{\begin{array}{c}{G}_{day}=0.1\ast {R}_{n}\\ {G}_{night}=0.5\ast {R}_{n}\end{array}\right\}$$

where the soil heat flux is considered to be 10% of the net radiation (*R*_*n*_) during the day and 50% during the night^6^ because the night-time heat flux is negative (upward). Note that we use the net solar radiation (*R*_*s*_) to define daytime and night-time periods for each day for each pixel.

Note that negative (night-time) values computed by our method have not been changed to zero in hPET, as is often done, since these values may be useful to some scientists to explore condensation processes. Many users of hPET may want to convert negative values to zero. The resulting hourly, 0.1° gridded dataset (hPET) was then aggregated by summation to daily timescales to obtain the daily dataset (dPET). Both datasets have been uploaded to a public data server for download (see below).

## Data Records

### Data file path

The initial temporal domain for hPET and dPET data is 1981–2019, but the dataset will be updated annually in January as new output data become available from ERA5-Land. These 0.1° datasets^40^ are located on a public data server at 10.5523/bris.qb8ujazzda0s2aykkv0oq0ctp.

### Data file format

The data within hPET and dPET are arranged on a yearly basis for the entire globe, where each netCDF (****.nc) filename begins with its year, followed by either ‘hourly_pet’ or ‘daily_pet’. For example, the global hPET dataset for 2019 is contained within the file called ‘2019_hourly_pet.nc’. The total sizes of individual (annual) files for these datasets are ~55GB for hPET and ~2.2GB for dPET. The total size of both datasets for 1981–2019 is ~2.2TB.

### File structure

Each file of hPET and dPET contains four variables (time, latitude, longitude, pet), as per Table 1. All times are in Coordinated Universal Time (UTC). Note that the first year of the hourly dataset, 1981, is missing its first hourly value for 00:00, so this year’s file contains only 8759 values for hPET. Latitude is given from 90° to −90° with 0.1° horizontal resolution (90°, 89.9°, 89.8°, …, −89.8°, −89.9°, −90°), and longitude ranges from −180° to 180° (−180°, −179.9°, −179.8°, …, 179.8°, 179.9°, 180°).

**Table 1:** Variables, values, and ranges for hPET and dPET; times are in UTC.

Variable name	Number of values		Value range	
	*hourly*	*daily*	*hourly*	*daily*
time	8760 per year 8784 (leap years)	365 per year 366 (leap years)	00:00, 01/01/1981 to 23:00, 31/12/2019	01/01/1981 to 31/12/2019
latitude	1801	1801	90° to −90°	90° to −90°
longitude	3600	3600	−180° to 180°	−180° to 180°
pet	8760 per year 8784 (leap years)	365 per year 366 (leap years)	00:00, 01/01/1981 to 23:00, 31/12/2019	01/01/1981 to 31/12/2019

## Technical Validation

After creating hPET, we investigated how our new dataset compares with existing PET products based on Priestley-Taylor (PT) and Penman-Monteith (PM) methods, which are in common use. Our objective here was not to present a performance hierarchy of PET datasets nor to identify which dataset might be better for a specific application. Each of the following datasets uses different definitions of PET and are therefore based on different formulations. Therefore, we make these PET comparisons to provide context about where our dataset fits in with existing ones generated at different spatial and temporal scales and by different methods. Specifically, we compare hPET to two other PET datasets based on the Penman-Monteith (PM) and two PET datasets based on the Priestley-Taylor (PT) method (Table 2). The PT method contains a coefficient, ‘alpha’, that represents the ratio between PET and the equilibrium evaporation, and as such it condenses the information contained in the aerodynamic part of the Penman–Monteith equation. It is often assumed to take a value of 1.26, as reported by Priestley and Taylor^7^ for well-watered grasslands, but it may vary based on vegetation type and higher values have been reported in advection regions^41,42^. The CRU_TS4.03 (ref. ^43^) is based on the FAO ET_0_, and thus uses a PM formulation assuming certain reference crop characteristics. This formulation is thus analogous to that of hPET (see Eq. 1), and it does not require the estimation of aerodynamic resistance. The MOD16 PET dataset is based on the PM formulation^44^, which parameterizes the aerodynamic resistance based on leaf area index and temperature information. On the other hand, the Global Land Evaporation Amsterdam Model (GLEAM)^45^ is based on a PT equation in which the alpha is land cover dependent. In GLEAM interception loss is estimated independently from the PET and not included within the PET estimates. The PT-JPL model^46^ uses the PT approach to estimate potential evaporation. However, unlike GLEAM, it uses a constant alpha of 1.26 and incorporates the interception loss within the PET.

**Table 2:** List of datasets used for comparison.

Dataset Name	Spatial Resolution	Temporal Resolution	Temporal Domain	Method	Data Source
CRU_TS4.03 PET (CRU) (https://crudata.uea.ac.uk/cru/data/hrg/)	0.5°	1 month	1901–2018	PM	^43^
MOD16 PET (MOD16) (http://files.ntsg.umt.edu/data/NTSG_Products/MOD16/)	0.5°	8 days	2001–2020	PM	^67^
GLEAM3.3 PET (GLEAM) (https://www.gleam.eu/)	0.25°	1 day	1981–2018	PT	^41,45^
PT-JPL (http://josh.yosh.org/datamodels.htm)	0.5°	1 month	1986–1995	PT	^68^
hPET (10.5523/bris.qb8ujazzda0s2aykkv0oq0ctp)	0.1°	1 hour	1981–2019	PM	^40^

The PT formulation only considers the radiative component of the PM equation and therefore does not require the parameterization of aerodynamic resistance. This simplification results in an underestimation of PET when net radiation is either limited or is not the main source of energy for evaporation such as in high latitudes in winter time, or in deserts^47^. In those conditions, the PM equation, which still considers adiabatic sources of energy to drive evaporation, is usually regarded as a more accurate approach (e.g.^32^).

**Fig. 1:**
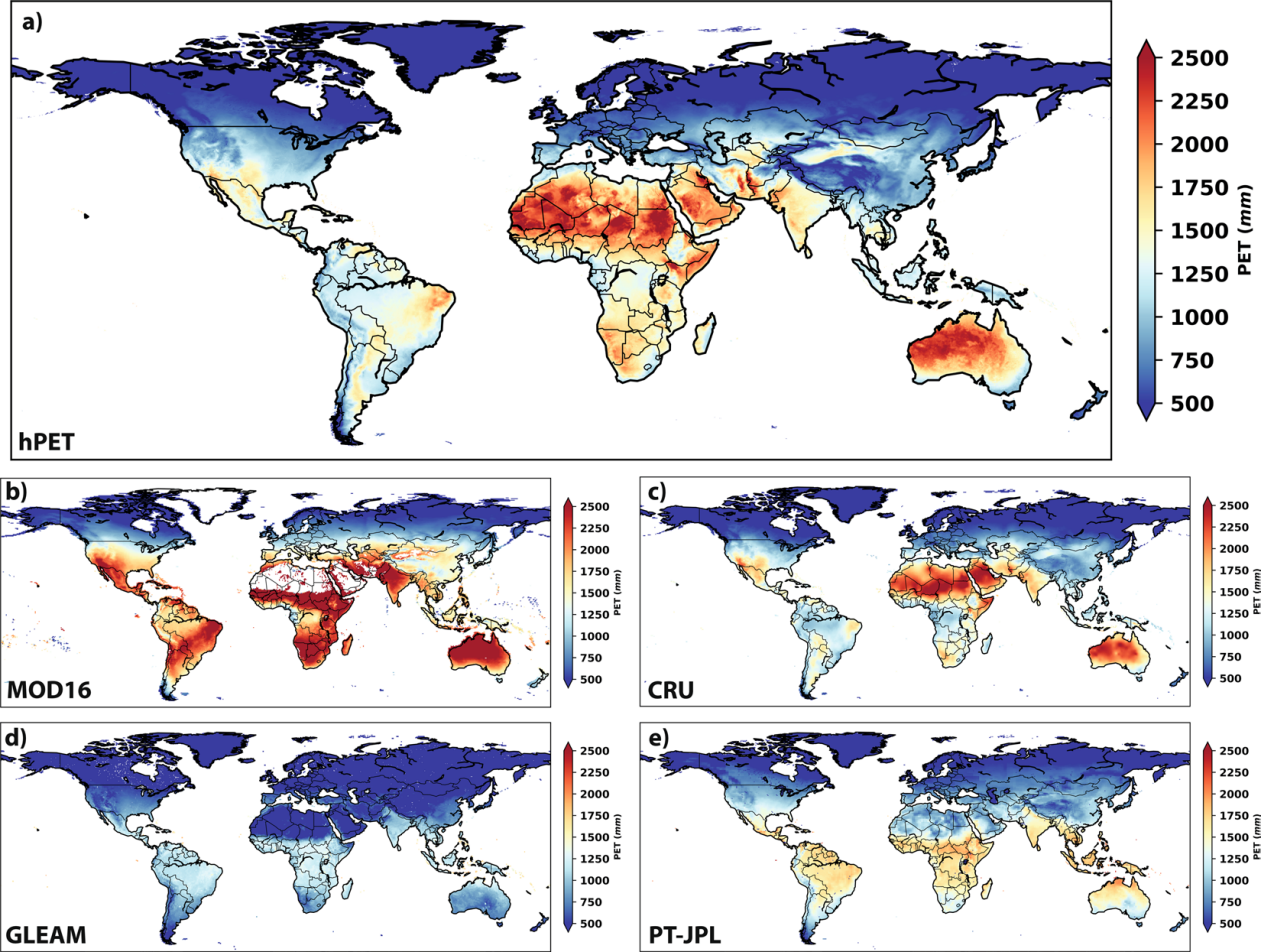
Climatology of PET for comparative datasets. Mean annual PET for hPET and each comparative PET dataset. The datasets, described in Table 2, are: (**a**) hPET, (**b**) MOD16, and (**c**) CRU, all developed via the PM method; and (**d**) GLEAM and (**e**) PT-JPL, developed via the PT method. Note: the MOD16 dataset has missing values, which appear as white (e.g., Sahara Desert).

Note that we have not included in our comparisons the inherent PET product output from ERA5-Land (a variable called PEV), since it was apparently not created by the conventional PM or PT approaches emphasized here. It is not clear how this variable is produced, and our analysis shows it to be inconsistent with the collection of PM and PT datasets compared here, with climatological values twice the maximum of all other datasets (scale bar maximum value of 5000 mm y^−1^ for PEV, compared to the of 2500 mm y^−1^ for all other datasets in Fig. 1). Therefore, we do not believe it represents a realistic and theoretically consistent estimate of PET for hydrological applications at the land surface.

The various PM and PT PET datasets compared here span different spatial and temporal resolutions, but they are broadly comparable (Fig. 1). The climatological comparisons, based on average annual PET for each 0.5° pixel over the global land surface, suggest that hPET has broadly similar geographical patterns to the other PM PET products, characterized by low PET values in northern latitudes due to low atmospheric energy availability and relatively high values in the equatorial regions and the global south (Fig. 1a–c). The hPET product compares closely with the CRU dataset (derived from gridded station data) as expected due to its common PET formulation, while the MOD16 dataset shows higher PET values across the global south and the tropics. In contrast, the two PT datasets (Fig. 1d,e) tend to express much lower values of PET across these same southern regions of the globe, particularly for GLEAM, with slightly higher values for the PT-JPL dataset. The values of GLEAM are particularly low in forests, since its PET is defined excluding canopy interception (which is computed separately), and in deserts. The latter is a common feature of PT models, and it arises from the relatively low values of net radiation in deserts due to the high shortwave and longwave outgoing radiation, and the strong sensitivity of PET to net radiation in the PT formulation.

**Fig. 2:**
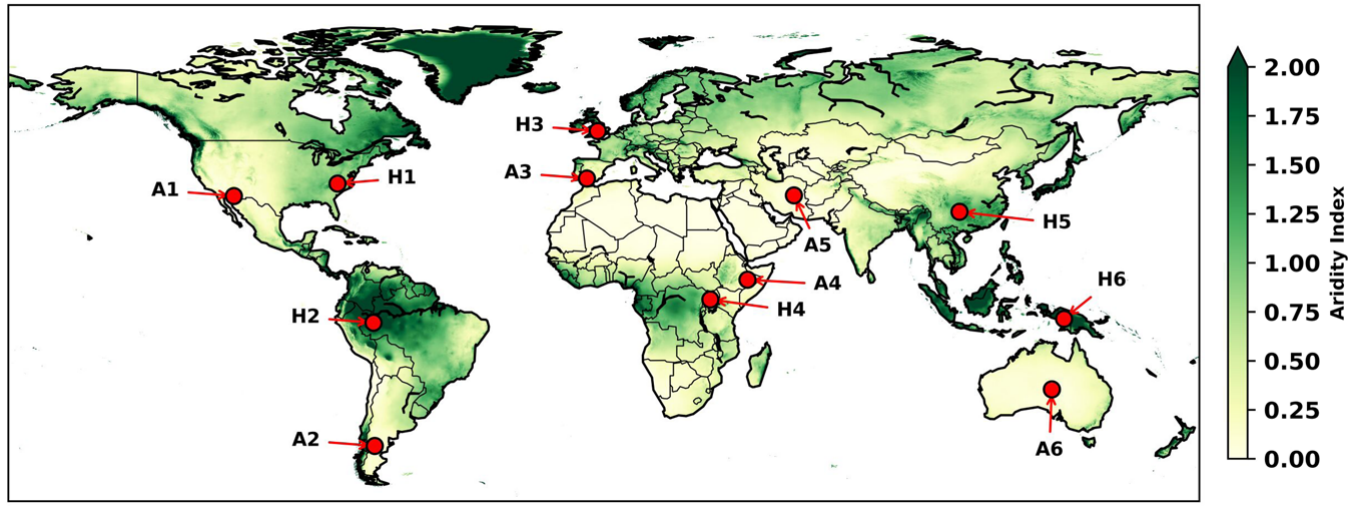
Locations for more detail with aridity background. Map of Aridity Index^48^ (precipitation/PET) for humid (H1, H2, etc.) and arid (A1, A2, etc.) sites selected for seasonal comparisons of different datasets and to show diurnal cyclicity within hPET for contrasting climatic regions within each continent (excluding Antarctica).

**Fig. 3:**
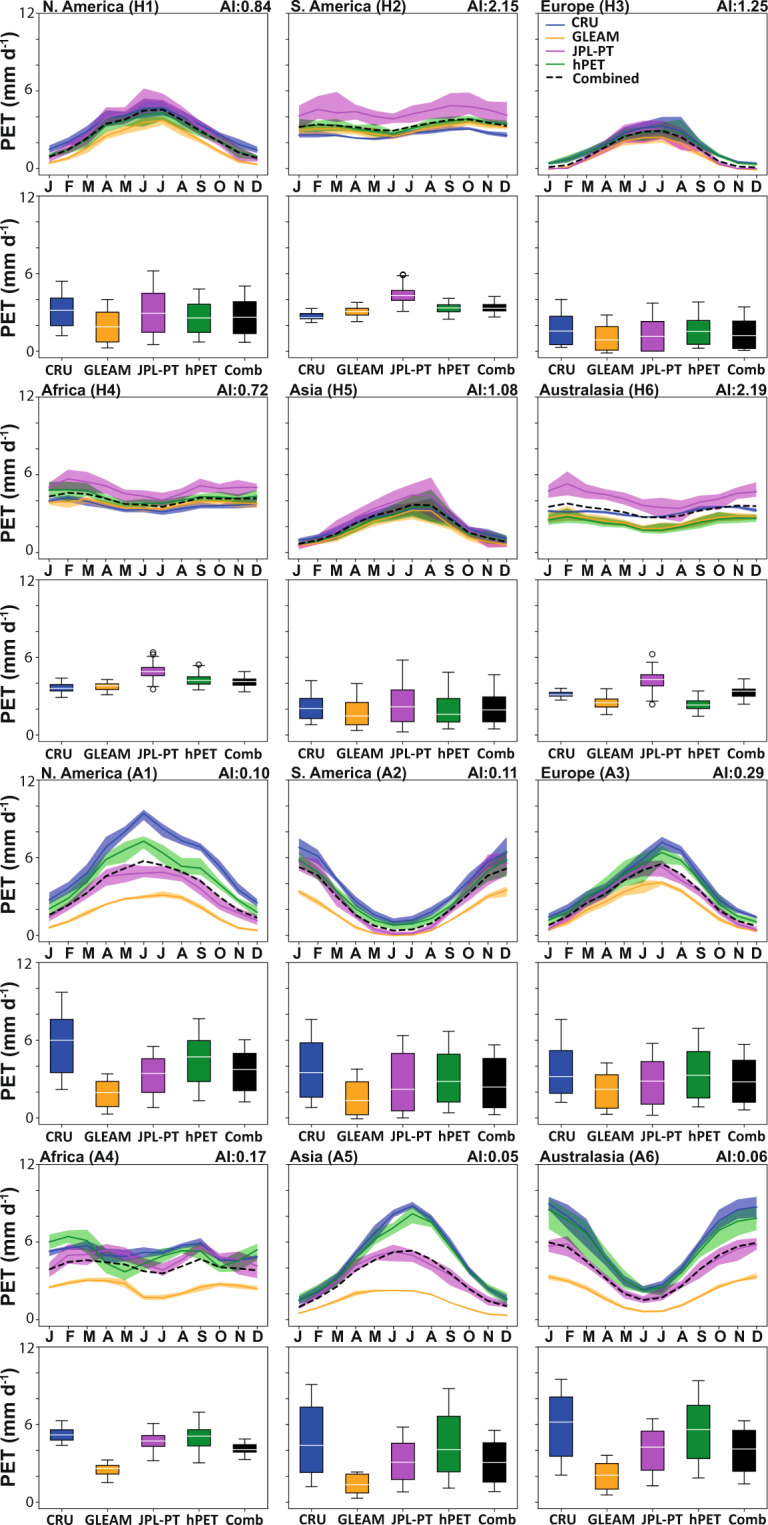
Seasonal variations in PET for comparative datasets. Monthly distributions of PET for hPET and comparative datasets over the temporal domains listed in Table 2. Distributional medians are shown as white horizontal lines. Sample PET data, aggregated to common spatial (0.5°) and temporal (1 month) resolutions, separated according to humid versus arid locations on each study continent (N. America, S. America, Europe, Africa, Asia, Australasia), as shown in Fig. 2. We show comparisons for 1986–1995, corresponding to the temporal domain of PT-JPL (note: some datasets were excluded from comparisons due lack of data for relevant temporal domains). Aridity Index (AI) values (from Fig. 2) are listed next to the site labels for context. Monthly ensemble seasonal distributions of PET are shown (above), as well as boxplots (below) that summarize all the data comparing all values. The datasets used for comparison are listed in Table 2. Note that we also show boxplots for ensemble mean distributions of PET from CRU, MOD16, GLEAM, and PT-JPL (as relevant) for each temporal period (in black and labeled ‘Combined’).

**Fig. 4:**
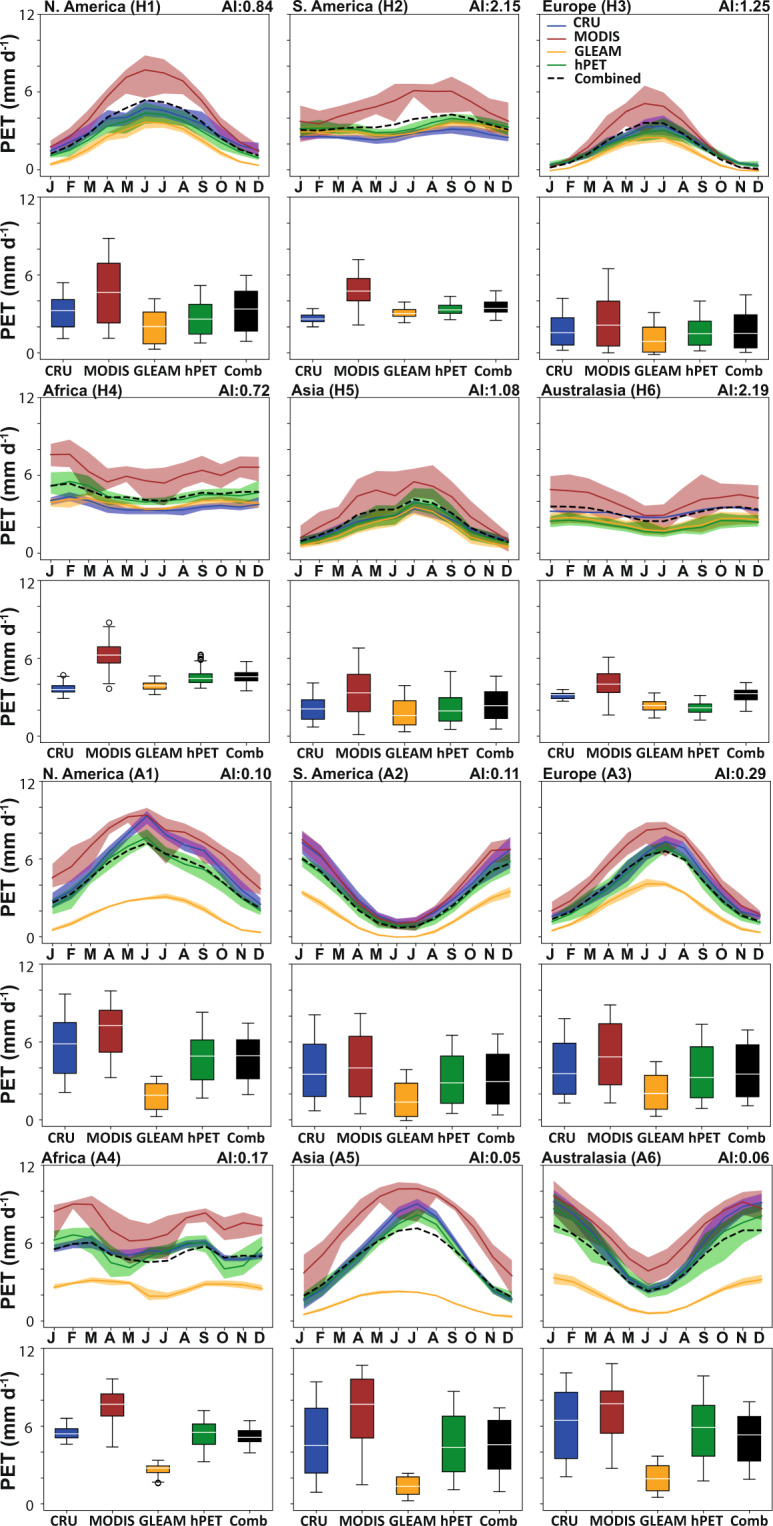
Seasonal variations in PET for comparative datasets. Monthly distributions of PET for hPET and comparative datasets over the temporal domains listed in Table 2. Distributional medians are shown as white horizontal lines. Sample PET data, aggregated to common spatial (0.5°) and temporal (1 month) resolutions, separated according to humid versus arid locations on each study continent (N. America, S. America, Europe, Africa, Asia, Australasia), as shown in Fig. 2. We show comparisons for 2001–2018, corresponding to most of the temporal domain of MOD16 (note: some datasets were excluded from comparisons due lack of data for relevant temporal domains). See caption for Fig. 3 for other relevant information.

**Fig. 5:**
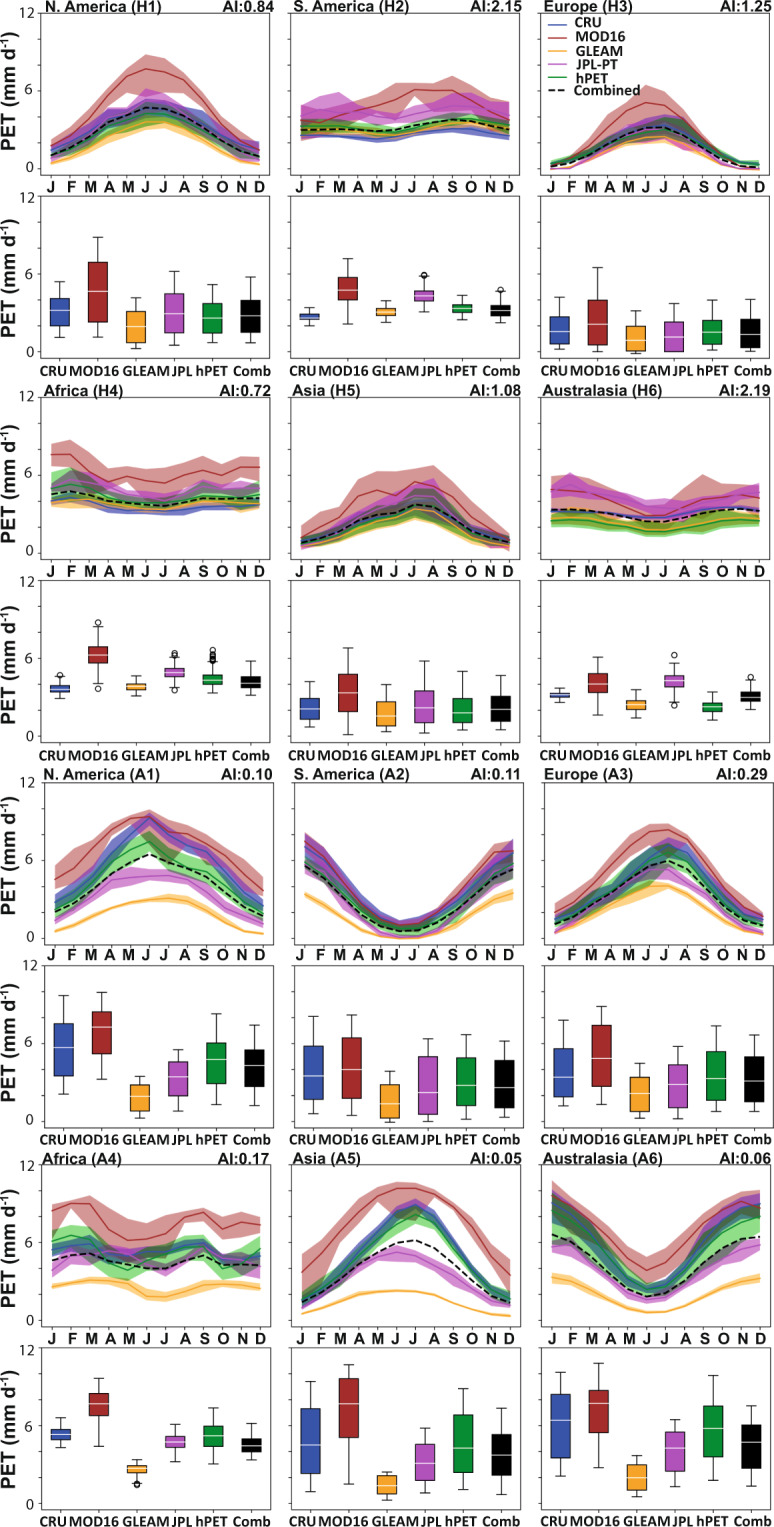
Seasonal variations in PET for comparative datasets. Monthly distributions of PET for hPET and comparative datasets over the temporal domains listed in Table 2. Distributional medians are shown as white horizontal lines. Sample PET data, aggregated to common spatial (0.5°) and temporal (1 month) resolutions, separated according to humid versus arid locations on each study continent (N. America, S. America, Europe, Africa, Asia, Australasia), as shown in Fig. 2. We show comparisons for 1981–2018, corresponding to most of the temporal domain of hPET (note: some datasets were excluded from comparisons due lack of data for relevant temporal domains). See caption for Fig. 3 for other relevant information.

We also assessed the seasonal cycles for each of these datasets and show results for a range of sites across the globe to sample a range of humid and arid conditions across all continents (Fig. 2), classified by the Aridity Index^48^ (precipitation/PET). For comparison, we aggregated all datasets to common spatial (0.5°) and temporal (monthly) resolutions. Figures 3–5 show the mean monthly values, the distributions of all PET datasets calculated over the time span of each individual dataset, and ensemble means of all comparative datasets. We show the temporal domain (up to and inclusive of 2018) for each dataset (where applicable) common to PT-JPL (Fig. 3); MOD16 (Fig. 4); and hPET (Fig. 5).

Generally, we see more differences between PET datasets for arid regions compared to humid regions (Figs. 3–5), which largely reflects differences in the method of PET formulation. We also observe good seasonal correspondence between hPET and CRU across all regions and a notable lack of correspondence with the PT datasets, and particularly GLEAM, due to the aspects discussed above (Figs. 3–5). More importantly, hPET seems to have a seasonal and overall distribution that is broadly similar to that of the ensemble mean of comparative datasets (‘Combined’), for both arid and humid regions across the globe (Figs. 2–5). There are notable exceptions for the arid site in Asia (A5) for the PT-JPL time domain (Fig. 3) and the humid site in Asia (H5) for the hPET domain (Fig. 5), both of which show poor correspondence between the ensemble (black) and hPET (green) most markedly expressed for the summer months. We also note that there is generally closer agreement between all datasets for the PT-JPL time domain (Fig. 3), compared to the others that capture the more recent period.

This evaluation demonstrates that hPET reproduces an overall pattern of global PET variability in a manner consistent with existing datasets, while simultaneously bringing additional insight into sub-daily and local variability. It is worth reiterating that we do not present these datasets comparisons to suggest any hierarchy, but merely to highlight how they differ for different regions of the globe and over seasonal timescales. The fact that PET is a notional concept, and that different datasets follow different definitions of this variable, prevents us from making statements of comparative data quality. Nonetheless, the higher temporal and spatial resolutions, as well as the robustness of the seasonal and geographical patterns, indicate that hPET is a valuable addition to the current palette of available PET products.

**Fig. 6:**
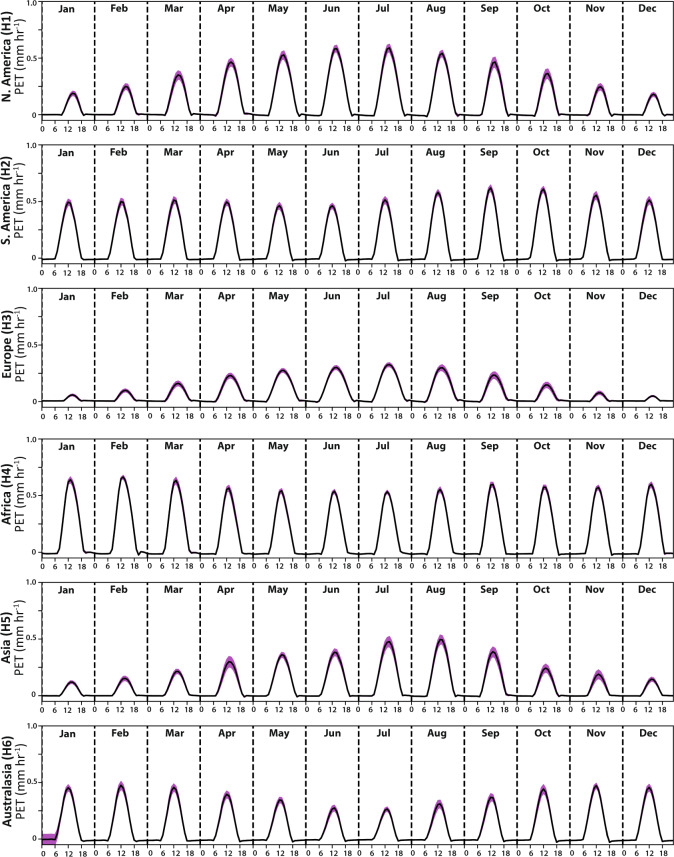
Diurnal cycles of hPET for humid regions. Diurnal cycle of hPET for humid regions on each continent for each month of the year, where each panel refers to a location shown in Fig. 2 (indicated on each y-axis). Pink shading indicates the standard deviation around the diurnal values of hPET based on the multi-decadal time series.

**Fig. 7:**
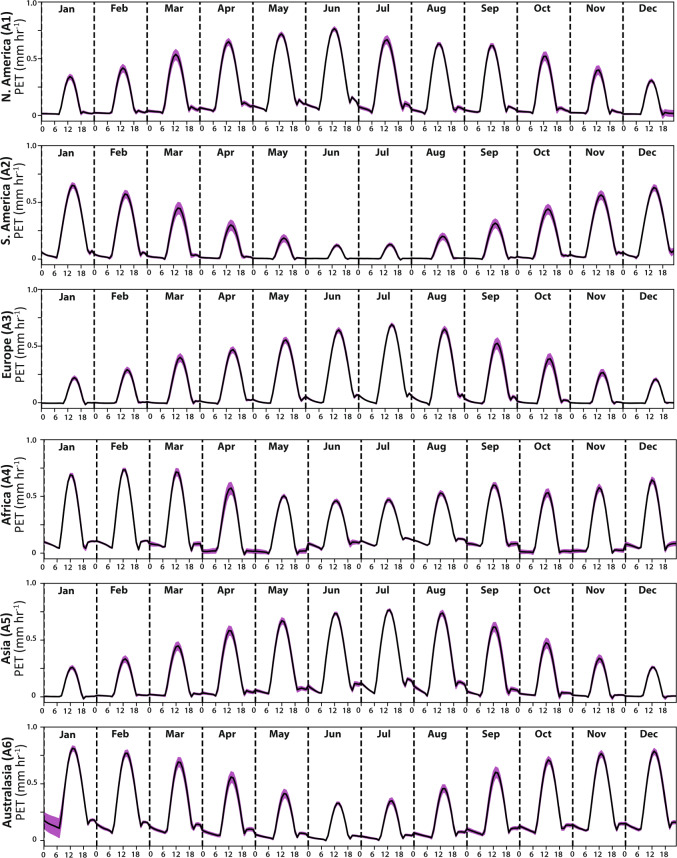
Diurnal cycles of hPET for arid regions. Diurnal cycle of hPET for arid regions on each continent for each month of the year, where each panel refers to a location shown in Fig. 2 (indicated on each y-axis). Pink shading indicates the standard deviation around the diurnal values of hPET based on the multi-decadal time series.

Next, we investigated hPET’s ability to capture diurnal cyclicity of PET across the globe, since the sub-daily resolution is an important feature of the new dataset. As expected, the ensemble diurnal cycles in hPET show values peaking just after noon for most months of the year at all our selected locations of the globe (Figs. 6 and 7), representing the increasing evaporative demand in daylight hours. We also spot clear differences in diurnal peaks between regions, for example, for humid regions of Africa and Europe (Fig. 6), and recognize that the magnitude of the peaks varies seasonally depending on the strength of solar radiation (e.g., contrasts between arid regions of North America and South America, Fig. 7). Finally, we observe that most of the variability in these monthly-mean diurnal cycles occurs around the daily peak.

Finally, we offer three examples of how hPET could be useful for a range of applications. We focus on the utility of hPET on topics of rainfall intensity, ecohydrology, and drought propagation. First, rainstorms have intensities that are dependent on the moisture in the atmosphere, which is in turn dependent on both moisture supply from land and evaporative demand in the atmosphere. Rainfall intensity is changing with shifts in global atmospheric temperatures and is highly sensitive to the short-term variations of energy supply and atmospheric water vapor demand^49–51^. Recent research has shown that the Clausius-Clapeyron relation between air temperature and moisture-holding capacity has different scaling relationships for different regions and that it may be most easily detected at high temporal resolution^52,53^. Our new hPET dataset shows promise for untangling this problem through analysis of high-resolution PET (hPET) alongside high-resolution rainfall data and/or by using these data to drive models to explore the relationships between evaporative demand and rainfall. Furthermore, hPET may be used as an atmospheric energy layer within stochastic rainstorm generation models that simulate precipitation fields with varying intensity^16,22,54^. The use of such high-resolution rainfall generators alongside datasets such as hPET will provide better constraints on land surface hydrology (e.g., cycles of wetting and drying) spanning diurnal to multidecadal variations.

Second, hPET could be useful in the analysis of the control of atmospheric demand for water on transpiration and plant water stress^25,55,56^. Since transpiration is downregulated by the high atmospheric demand for water causing plant stomata to close, PET could be an important determinant of plant water stress. Having atmospheric demand data at sub-daily resolutions is critical to disentangle the stomatal effect from the effect of low soil water availability, which does not exhibit a daily cycle. While root zone water availability may or may not increase in the future for many regions^57,58^, the atmospheric demand is increasing in most places^55,59^. The new hPET dataset enables the analysis of historical evaporative demand alongside plant-level or canopy-level responses to water availability over diurnal cycles, which could aid in understanding how vegetated environments might evolve under climate change that especially impacts atmospheric evaporative demand^56,60^.

Third, the propagation of meteorological drought into agricultural and hydrological droughts is of critical importance to human society, especially in dryland regions^61–63^, and future droughts may threaten broad areas of the globe^64^. Droughts develop due to progressive losses of water through atmospheric evaporative demand without replacement from rainfall. Irrespective of trends or changes in rainfall, droughts may arise solely due to shifts in atmospheric demand for water vapor^55,65^, and there are ongoing debates about whether we should expect increasing drought hazard/risk in the future^34,66^. Nevertheless, onset, propagation, and severity of droughts are closely tied to the complex feedbacks between precipitation, PET, soil moisture, and vegetation, which may develop and intensify over diurnal cycles, yet the detailed mechanisms are not well understood^55^. Therefore, hPET could be a valuable tool for retrospective analysis of past droughts via data and/or models, to develop and refine drought metrics, and also as cautionary guidance for what we may expect in future drought scenarios for specific regions.

These examples illustrate that hPET will be a useful new PET product for analysis of the water balance at high spatial and temporal resolutions. We have shown its ability to capture: 1) broad geographical patterns of PET climatology comparable to similar products; 2) seasonal variations of PET across the globe, broadly comparable to an amalgamation of comparable datasets, and 3) diurnal cyclicity required to resolve water balance changes over fine temporal resolutions. We have also highlighted some examples of how hPET could be used, out of a wider range of potential applications related to the assessment of climatic impacts at the land surface.

## Data Availability

The codes we developed for computing hPET and dPET are available at https://github.com/Dagmawi-TA/hPET. In addition, we are providing users with a simple Python script to enable easy access to specific parts of the complete hPET and dPET datasets, based on user needs. This latter script allows the user to specify a geographical box and a selection of years for which data are required. The code then accesses the raw data files and downloads the data for relevant temporal and spatial domains from an open-access data server (10.5523/bris.qb8ujazzda0s2aykkv0oq0ctp). Since the data are freely available, one can download the script and run it on a local machine to download all or a portion of the data. The scripts are all documented, and readme files are provided from the relevant repository.
